# Neutrophil Immunomodulatory Activity of Nerolidol, a Major Component of Essential Oils from *Populus balsamifera* Buds and Propolis

**DOI:** 10.3390/plants11233399

**Published:** 2022-12-06

**Authors:** Igor A. Schepetkin, Gulmira Özek, Temel Özek, Liliya N. Kirpotina, Polina I. Kokorina, Andrei I. Khlebnikov, Mark T. Quinn

**Affiliations:** 1 Department of Microbiology and Cell Biology, Montana State University, Bozeman, MT 59717, USA; 2 Department of Pharmacognosy, Faculty of Pharmacy, Anadolu University, Eskisehir 26470, Turkey; 3 Kizhner Research Center, Tomsk Polytechnic University, Tomsk 634050, Russia

**Keywords:** calcium flux, chemotaxis, essential oil, nerolidol, neutrophil, *Populus balsamifera*, propolis

## Abstract

Propolis is a resinous mixture of substances collected and processed from various botanical sources by honeybees. Black poplar (*Populus balsamifera* L.) buds are one of the primary sources of propolis. Despite their reported therapeutic properties, little is known about the innate immunomodulatory activity of essential oils from *P. balsamifera* and propolis. In the present studies, essential oils were isolated from the buds of *P. balsamifera* and propolis collected in Montana. The main components of the essential oil from *P. balsamifera* were *E*-nerolidol (64.0%), 1,8-cineole (10.8%), benzyl benzoate (3.7%), α-terpinyl acetate (2.7%), α-pinene (1.8%), o-methyl anisol (1.8%), salicylaldehyde (1.8%), and benzyl salicylate (1.6%). Likewise, the essential oil from propolis was enriched with *E*-nerolidol (14.4%), cabreuva oxide-VI (7.9%), α-bisabolol (7.1%), benzyl benzoate (6.1%), β-eudesmol (3.6%), T-cadinol (3.1%), 2-methyl-3-buten-2-ol (3.1%), α-eudesmol (3.0%), fokienol (2.2%), nerolidol oxide derivative (1.9%), decanal (1.8%), 3-butenyl benzene (1.5%), 1,4-dihydronaphthalene (1.5%), selina-4,11-diene (1.5%), α-cadinol (1.5%), linalool (1.4%), γ-cadinene (1.4%), 2-phenylethyl-2-methyl butyrate (1.4%), 2-methyl-2-butenol (1.3%), octanal (1.1%), benzylacetone (1.1%), and eremoligenol (1.1%). A comparison between *P. balsamifera* and propolis essential oils demonstrated that 22 compounds were found in both essential oil samples. Both were enriched in *E*-nerolidol and its derivatives, including cabreuva oxide VI and nerolidol oxides. *P. balsamifera* and propolis essential oils and pure nerolidol activated Ca^2+^ influx in human neutrophils. Since these treatments activated neutrophils, the essential oil samples were also evaluated for their ability to down-regulate the neutrophil responses to subsequent agonist activation. Indeed, treatment with *P. balsamifera* and propolis essential oils inhibited subsequent activation of these cells by the *N*-formyl peptide receptor 1 (FPR1) agonist *f*MLF and the FPR2 agonist WKYMVM. Likewise, nerolidol inhibited human neutrophil activation induced by *f*MLF (IC_50_ = 4.0 μM) and WKYMVM (IC_50_ = 3.7 μM). Pretreatment with the essential oils and nerolidol also inhibited human neutrophil chemotaxis induced by *f*MLF, again suggesting that these treatments down-regulated human neutrophil responses to inflammatory chemoattractants. Finally, reverse pharmacophore mapping predicted several potential kinase targets for nerolidol. Thus, our studies have identified nerolidol as a potential anti-inflammatory modulator of human neutrophils.

## 1. Introduction

Propolis is a resinous beehive product that is collected by bees from plant exudates and is used to protect and maintain hive homeostasis [1,2]. Propolis has been used by humans therapeutically to treat inflammation and infectious diseases [3,4,5,6,7,8]. Numerous studies have revealed a range of biological activities of propolis, including antibacterial, antifungal, antiviral, anticancer, anti-inflammatory, immunomodulatory, and antioxidant activities [9,10,11,12,13,14,15]. Although the anti-inflammatory and immunomodulatory activities of propolis are well established (reviewed in [16,17]), it is still unclear how propolis components could contribute to all of these biological properties. To date, over 800 compounds have been reported to be present in propolis, including alcohols, acids and their esters, benzofuranes, benzopyranes, chalcones, flavonoids and their esters, glycosides, glycerol and its esters, lignans, phenylpropanoids, steroids, terpenes, and terpenoids [2,18].

The sources of propolis are mainly plant exudates from bark and buds [19], and the main source of propolis in the northern temperate zone is poplar buds. Indeed, balsam poplar (*Populus balsamifera* L.) buds are polyphenol-rich, with a chemical composition close to that of propolis [20]. *P. balsamifera* buds have been used traditionally by American Indians to treat various skin problems, such as psoriasis, eczema, sores, and inflamed wounds [21]. Additionally, the Cherokee people have used a tincture of poplar buds for chronic rheumatism and as a gastrointestinal aid [21]. Extracts from poplar buds were also reported to exhibit anti-inflammatory and immunomodulatory activities [22,23,24].

Poplar buds are a rich source of essential oils [25,26], and essential oils from various plant species are reported to exhibit antimicrobial, anticancer, anti-inflammatory, and immunomodulatory effects (reviewed in [27,28,29]). Recently we found that essential oils from *Artemisia kotuchovii* Kupr., *Ferula akitschkensis* B.Fedtsch. ex Koso-Pol., *Ferula iliensis* Krasn. ex Korovin, *Hypericum perforatum* L., *Grindelia squarrosa*, *Rhododendron albiflorum* Hook., and *Juniperus* and *Artemisia* spp. can modulate human neutrophil functions [30,31,32,33,34,35,36]. Moreover, essential oils and some of their compounds (e.g., monoterpenes, sesquiterpenes) are reported to modulate humoral and cellular immune responses [37,38,39,40,41,42]. Thus, we hypothesized that essential oils from *P. balsamifera* buds and propolis collected nearby might be a novel source of anti-inflammatory compounds.

Neutrophils are the most abundant immune cells, and their activation is critical for innate immunity and initiation of the inflammatory process [43]. These cells rapidly respond to infection and injury to establish the first line of defense through multiple mechanisms, such as reactive oxygen species production, phagocytosis, and microbicidal enzyme secretion [44,45]. On the other hand, unregulated or chronic neutrophil activation through G-protein-coupled receptors (GPCRs) could lead to host tissue damage [46]. Therefore, GPCRs or their downstream molecules are major targets for inhibiting uncontrolled neutrophil activation [47], and numerous natural products, including essential oils, are shown to exhibit neutrophil immunomodulatory activity [30,31,32,33,34,35,36,47,48].

In the present study, essential oils were isolated from the buds of *P. balsamifera* and propolis collected in Montana and analyzed for their chemical compositions and innate immunomodulatory activities. These essential oils potently inhibited intracellular Ca^2+^ mobilization ([Ca^2+^]_i_) in human neutrophils. Furthermore, nerolidol, which was present at high levels in both essential oil samples, also inhibited human neutrophil functional responses. Thus, nerolidol is likely one of the main active components in these essential oils. Given the critical role of neutrophils in inflammation, these data support the possibility that nerolidol could be considered in the development of new anti-inflammatory agents.

## 2. Results and Discussion

### 2.1. Composition of Essential Oil from P. balsamifera Buds and Propolis

Essential oils were obtained from the buds of *P. balsamifera* (designated by PBO) and propolis (designated by PRO) for subsequent phytochemical and biological characterization. The yields (*v*/*w*) of the essential oils from buds of *P. balsamifera* and propolis were 3.5% and 0.2%, respectively, and the chemical composition of these essential oils was evaluated using simultaneous GC-FID and GC/MS.

A total of 52 compounds were identified in PBO, accounting for 98.4% of the essential oils, with *E*-nerolidol (64.0%), 1,8-cineole (10.8%), benzyl benzoate (3.7%), α-terpinyl acetate (2.7%), α-pinene (1.8%), o-methyl anisol (1.8%), salicylaldehyde (1.8%), and benzyl salicylate (1.6%) being the major compounds present at >1%. The major classes of compounds in PBO were oxygenated sesquiterpenes (65.4%) and oxygenated monoterpenes (15.6%) (Table 1).

The major compounds identified in PRO were *E*-nerolidol (14.4%), cabreuva oxide-VI (7.9%), α-bisabolol (7.1%), benzyl benzoate (6.1%), β-eudesmol (3.6%), T-cadinol (3.1%), 2-methyl-3-buten-2-ol (3.1%), α-eudesmol (3.0%), fokienol (2.2%), nerolidol oxide derivative (1.9%), decanal (1.8%), 3-butenyl benzene (1.5%), 1,4-dihydronaphthalene (1.5%), selina-4,11-diene (1.5%), α-cadinol (1.5%), linalool (1.4%), γ-cadinene (1.4%), 2-phenylethyl-2-methyl butyrate (1.4%), 2-methyl-2-butenol (1.3%), octanal (1.1%), benzylacetone (1.1%), and eremoligenol (1.1%). Overall, the major classes of compounds in PRO were oxygenated sesquiterpenes (48.5%) and sesquiterpene hydrocarbons (14.1%) (Table 1).

A comparison of PBO and PRO demonstrated that 22 compounds (from 107 identified compounds) were present in both essential oil samples. The chemical composition of the PBO from Montana was significantly different than essential oils extracted from poplar buds in other locations. The main distinguishing feature of PBO was the extremely high amount of *E*-nerolidol (64%), whereas essential oils isolated from Canadian *P. balsamifera* buds were much lower by far (4.0–5.6%) [49]. The Canadian balsam poplar bud’s oils (spring, fall) were characterized by high levels of the monocyclic sesquiterpene alcohol α-bisabolol (18.2–67.7%), as well as γ-cadinene (1.5–4.0%), δ-cadinene (2.2–10.0%), and minor amounts of 1,8-cineole [49]. In contrast, α-bisabolol was absent, γ- and δ-cadinene were present in trace amounts, and large amounts of 1,8-cineole were found (10.8%) in PBO samples. The volatiles extracted from Polonian balsam poplar buds were characterized by high levels of the tertiary bicyclic alcohols guaiol (13.2%) and bulnesol (9.7%), as well as significant amounts of α-, β-, and γ-eudesmol (9.1%) [50]. In contrast, these constituents were not present in PBO.

Previously, nerolidol was identified in essential oils extracted from propolis collected in the Canary Islands (3.2–11.0%) and Brazil (6.6–17.1%) [51,52,53,54], which were a little lower or similar to the levels identified in PRO. Essential oil/volatiles present in propolis from different countries suggest a wide diversity in chemical composition. It is likely that this diversity reflects the phytogeographic characteristics of the bee harvesting site, which makes the comparison of different samples of this natural product difficult [55]. Volatile compounds of propolis consisted mainly of terpenoids and aromatic compounds with low molecular masses. Monoterpenes dominated the essential oils of Greek propolis, including α-pinene (7.9–45.8%) and *trans*-β-terpineol (2.2–6.6%), with lower levels of sesquiterpenes, such as α-muurolene (1.5–5.0%), δ-cadinene (0.3–8.4%), cedrol (4.3–6.3%), and α-, β-, and γ-eudesmol (12.1%, 4.7%, and 4.0%, respectively) [56]. Bulgarian propolis essential oils were characterized by a high content of sesquiterpenes, β-eudesmol (8.8%), δ-cadinene (5.3%), and γ-muurolene (4.7%) [57]. Brazilian propolis samples contained prenyl and diprenyl acetophenones (3.6–8.2% and 1.7–11.1%, respectively), while the sesquiterpenes were represented by (2*Z*, 6*E*)-farnesol (6.1–17.4%), sesquiterpene alcohol [M ^+^ =220] (12.9%), δ-cadinene (0.7–3.3%), and ledol (0.1–5.7%) [58]. The major volatile components identified in Dalmatian propolis were terpenes (30%), while benzyl alcohol, benzoic acid, and benzyl benzoate were predominant (49%) in propolis from Slavonia [59]. Recently, propolis essential oil samples from acaricide-treated and untreated beehives in southern Portugal were found to contain thymol (0.1–78.8%), viridiflorol (2.7–37.9%), guaiol (0.1–5.0%), ledol (0.1–4.2%), and β-eudesmol (0.1–3.4%) [55]. These essential oils also contained sesquiterpenoids of the drimane group, such as ambroxide (0.4–5.5%), 8-*epi*-13-*nor*-ambreinolide (t-3.8%), 13-*nor*-ambreinolide (0.1–2.7%), 6-acetoxy-11-*nor*-drim-7-en-9-one (0.6–0.8%), as well as labdane diterpenoids, labd-7-en-15-ol (0.1–15.1%), labd-8-en-15-ol (0.1–3.3%), 15-*nor*-labdan-8-ol (1.4–10.8%), lab-8(17)-en-15-ol (0.1–3.2%), and manoyl oxide (0.1–0.4%). In contrast, PRO collected in Montana did not contain diterpenes or drimane group sesquiterpenes. By comparison with the other propolis essential oils analyzed, it is evident that PRO has a unique compositional profile due to the presence of nerolidol derivatives and benzoic acid derivatives.

Nerolidol (3,7,11-trimethyl-1,6,10-dodecatrien-3-ol), an acyclic sesquiterpene, is also known as peruviol and penetrol. It has been reported as a major constituent (up to 90%) in essential oils of many plant species, such as *Baccharis dracunculifolia* DC (Asteraceae), *Melaleuca leucadendra* L. (Myrtaceae), *Piper calussenianum* (Piperaceae) [60,61,62,63,64], and fungal extracts [65]. Because of the geometric and asymmetric center in two carbons, nerolidol has four different isomeric forms: *S*- and *R*-enantiomers and geometric *E*- and *Z*-isomers [66,67,68]. Although there is not much reported on the enantiomeric status of nerolidol in essential oils, (3*S*,6*E*)-nerolidol was identified in cabreuva oil from *Myrocarpus frondosus* and *M. fastigiatus* Allem and Peru balsam from *Myroxylon pereira* Klotzsch [69,70]. Likewise, the seed oils of *Aframomum dalzielii* Hutchinson, *A. letestuianum* Gagnepain, and *A. pruinosum* Gagnepain from Cameroon were characterized by a high content of (3*R*,6*E*)-nerolidol (>88.0%) [66].

**Table 1:** Chemical composition of essential oil (%) isolated from *P. balsamifera* buds (PBO) and propolis (PRO).

No	RRI	RRI ^@^	Compound	PBO	PRO	No	RRI	RRI ^@^	Compound	PBO	PRO
1	1032	1008–1039 ^a^	α-Pinene	1.8		55	1703	1629–1704 ^a^	Salicylaldehyde	1.8	
2	1035	1012–1039 ^a^	α-Thujene	0.3		56	1704	1655–1714 ^a^	γ-Muurolene	T	0.9
3	1048	1005–1075 ^b^	2-Methyl-3-buten-2-ol		3.1	57	1704	1682–1704 ^a^	γ-Curcumene		0.2
4	1057	1026–1088 ^b^	Toluene		0.7	58	1706	1659–1724 ^a^	α-Terpineol		0.5
5	1076	1043–1086 ^a^	Camphene	t		59	1709	1672–1718 ^a^	α-Terpinyl acetate	2.7	
6	1118	1085–1130 ^a^	β-Pinene	0.6		60	1719	1702–1708 ^b^	Zonarene		0.3
7	1132	1098–1140 ^a^	Sabinene	0.1		61	1726	1676–1726 ^a^	Germacrene D		0.2
8	1145	1100–1178 ^b^	Ethyl benzene		0.2	62	1737	1713–1748 ^a^	(*Z,E*)-α-Farnesene	0.2	
9	1174	1140–1175 ^a^	Myrcene	0.3	0.2	63	1740	1686–1753 ^a^	α-Muurolene	t	0.8
10	1176	1148–1186 ^a^	α-Phellandrene	t		64	1741	1698–1748 ^a^	β-Bisabolene		0.7
11	1188	1154–1195 ^a^	α-Terpinene	0.2		65	1742	1686–1743 ^a^	β-Selinene		0.4
12	1203	1178–1219 ^a^	Limonene	0.5	0.6	66	1744	1696–1748 ^a^	α-Selinene		0.7
13	1213	1186–1231 ^a^	1,8-Cineole	10.8	0.9	67	1744	1689–1771 ^a^	Benzyl acetate	0.1	0.1
14	1255	1222–1266 ^a^	γ-Terpinene	0.6		68	1755	1711–1756 ^a^	β-Curcumene		0.4
15	1268	1249–1266 ^b^	Prenyl acetate		0.1	69	1758	1714–1763 ^a^	(*E,E*)-α-Farnesene	0.7	0.2
16	1272	1240–1290 ^a^	Vinyl benzene		0.8	70	1771	1726–1773 ^a^	γ-Bisabolene		0.3
17	1280	1246–1291 ^a^	*p*-Cymene	0.7		71	1773	1722–1774 ^a^	δ-Cadinene	0.1	0.2
18	1290	1261–1300 ^a^	Terpinolene	0.1		72	1776	1735–1782 ^a^	γ-Cadinene	0.1	1.4
19	1296	1267–1312 ^a^	Octanal		1.1	73	1784	1763–1786 ^a^	(*E*)-α-Bisabolene		0.2
20	1348	1317–1357 ^a^	6-Methyl-5-hepten-2-one	0.1		74	1786	1743–1788 ^a^	*ar*-Curcumene	t	0.3
21	1369	-	3-Butenyl benzene ^#^		1.5	75	1798	1727–1809 ^a^	Methyl salicylate	0.2	
22	1371	1308–1328 ^b^	2-Methyl-2-butenol		1.3	76	1805	-	Nerolidol oxide der. *	1.0	1.9
23	1387	-	MOMP ^#^		0.3	77	1819	1815 ^b^	α-Cadinene		0.4
24	1388	1398 ^c^	DMNT ^#^	0.1		78	1825	1823 ^b^	Cabreuva oxide VI		7.9
25	1400	1390–1432 ^b^	o-Methyl anisole	1.8		79	1838	1784–1851 ^a^	2-Phenylethyl acetate		0.6
26	1400	1370–1414 ^a^	Nonanal		0.7	80	1853	1800–1853 ^a^	*cis*-Calamenene	t	0.9
27	1416	1397	1-Ethenyl-4-methyl benzene ^d^		0.3	81	1859	1837–1882 ^b^	Benzylacetone	0.1	1.1
28	1443	1425–1459 ^b^	2,5- Dimethylstyrene		0.1	82	1866	1842–1866 ^b^	Methyl hydrocinnamate		0.4
29	1450	1429–1481 ^a^	*trans*-Linalool oxide	0.1	0.7	83	1900	1900 ^e^	Nonadecane		0.3
30	1478	1410–1478 ^a^	*cis*-Linalool oxide		0.3	84	1902	1880–1908 ^b^	Benzyl isovalerate	0.1	
31	1493	1459–1500 ^a^	α-Ylangene	t	0.1	85	1932	1894–1937 ^b^	3-Methylbutyl benzoate	0.1	
32	1497	1462–1522 ^a^	α-Copaene	t	0.3	86	1941	1893–1941 ^a^	α-Calacorene		0.5
33	1506	1471–1516 ^a^	Decanal		1.8	87	1948	1988 ^f^	Nerolidol oxide I		tr
34	1538	1551 ^b^	*p*-Ethyl anisole *		0.1	88	1950	1950 ^b^	PEMB		1.4
35	1541	1481–1555 ^a^	Benzaldehyde	0.1	0.3	89	2025	2016 ^f^	Nerolidol oxide II		0.9
36	1553	1507–1564 ^a^	Linalool		1.4	90	2050	1995–2055 ^a^	*E*-Nerolidol	64.0	14.4
37	1565	1532–1570 ^a^	Linalyl acetate	0.7		91	2061	1986–2065 ^a^	4-Ethylguaiacol		0.4
38	1594	1559–1609 ^b^	*trans*-β-Bergamotene	t	0.2	92	2080	2019–2090 ^a^	Cubenol		0.5
39	1602	1582–1604 ^b^	MHDO		0.3	93	2088	2049–2088 ^b^	Methyl-o-anisate	0.2	
40	1605	1600–1642 ^b^	*epi*-Bicyclosesquiphellandrene	t		94	2156	2156 ^g^	α-Bisabolol oxide B		0.9
41	1608	1542–1628 ^b^	β-Copaene	t		95	2164	2170–2187 ^b^	Fokienol	0.4	2.2
42	1611	1564–1630 ^a^	Terpinen-4-ol	0.7		96	2179	2164–2210 ^b^	4-Ethylphenol		0.4
43	1616	1580–1616 ^a^	Hotrienol		0.6	97	2187	2151–2198 ^b^	T-Cadinol		3.1
44	1630	1609–1687 ^a^	Terpinen-4-yl acetate	0.6		98	2205	2204–2205 ^b^	Eremoligenol		1.1
45	1638	1548–1638 ^a^	β-Cyclocitral		0.3	99	2209	2151–2209 ^b^	T-Muurolol		0.7
46	1641	1583–1656 ^a^	Methyl benzoate	0.5		100	2214	-	α-Guaiol ^#^		0.8
47	1641	-	1,4-Dihydronaphthalene ^#^		1.5	101	2219	2150–2233 ^b^	δ-Cadinol		0.4
48	1661	1624–1668 ^a^	Alloaromadendrene		0.4	102	2232	2178–2234 ^a^	α-Bisabolol		7.1
49	1668	1627–1668 ^a^	(*Z*)-β-Farnesene	0.1	0.3	103	2250	2186–2250 ^a^	α-Eudesmol		3.0
50	1671	1607–1699 ^a^	Acetophenone		0.6	104	2255	2180–2255 ^a^	α-Cadinol		1.5
51	1677	1672–1692 ^b^	*epi*-Zonarene		0.1	105	2257	2196–2272 ^a^	β-Eudesmol		3.6
52	1685	1640–1706 ^a^	Ethyl benzoate	0.2		106	2655	2565–2655 ^a^	Benzyl benzoate	3.7	6.1
53	1688	1664–1688 ^b^	Selina-4,11-diene	0.3	1.5	107	2785	2760–2810 ^a^	Benzyl salicylate	1.6	
54	1694	1670–1694 ^b^	*p*-Vinylanisole		0.7						

Legend: The data are presented as relative % for each component identified. * Correct isomer not identified; ^#^ tentative identification from mass spectra similarity. All other compounds were identified by comparison with co-injected standards. RRI, relative retention index, calculated based on retention of *n*-alkanes; ^@^ RRI, reported in the literature ^a–g^ [71,72,73,74,75,76,77]. Trace amounts (t) were present at <0.1%. Nerolidol oxide derivative (7*S*, 10*S*,5*E*)-2,6,10-trimethyl-7,10-epoxy-2,5,11-dodecatriene); DMNT, 4,8-dimethyl-1,3,7-nonatriene; MHDO, 6-methyl-3,5-heptadien-2-one; MOMP, 3-methyl-2-oxo-methyl pentanoate; PEMB, 2-phenylethyl-2-methyl butyrate.

### 2.2. Effect of PBO, PRO, and Nerolidol on Neutrophil Ca^2+^ Influx

PBO and PRO were evaluated for their immunomodulatory effects on human neutrophils. Specifically, their effects on intracellular Ca^2+^ flux [Ca^2+^]_i_ were evaluated, which is a key component of neutrophil activation and function [78]. Treatment of neutrophils with either PBO or PRO activated human neutrophils, resulting in increased [Ca^2+^]_i_ (EC_50_ = 10.5 µg/mL and 18.3 µg/mL, respectively) (Table 2).

**Figure 1:**
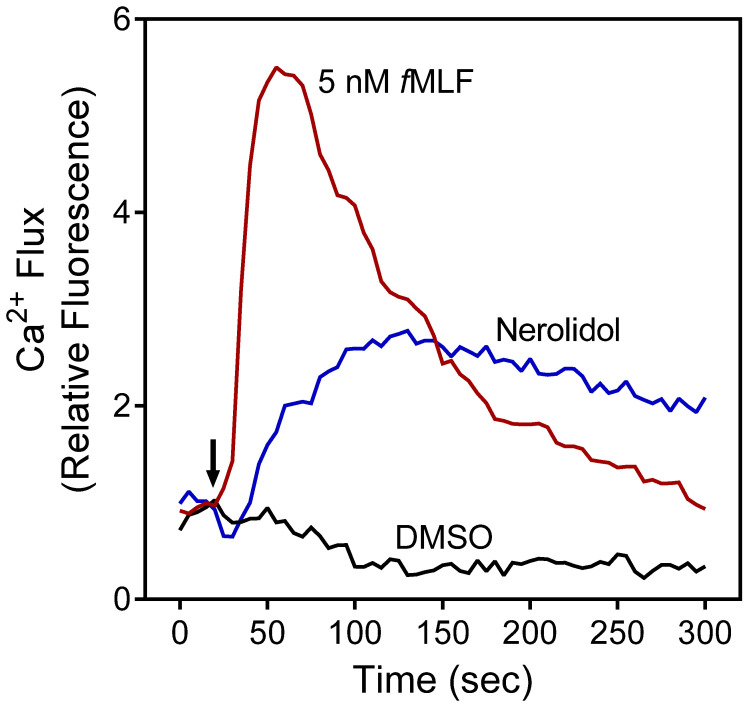
Effect of nerolidol on neutrophil [Ca^2+^]_i_. Human neutrophils were treated with 5 µM nerolidol, 5 nM *f*MLF (positive control), or 1% DMSO (negative control), and [Ca^2+^]_i_ was monitored for the indicated times (arrow indicates when treatment was added). Data are from one experiment that is representative of three independent experiments.

Analysis of the direct effect of nerolidol on human neutrophils showed that nerolidol (mixture of *E*/*Z* isomers) activated [Ca^2+^]_i_ with an EC_50_ = 0.8 µM, and a representative kinetic curve for neutrophil [Ca^2+^]_i_ induced by nerolidol is shown in Figure 1.

**Figure 2:**
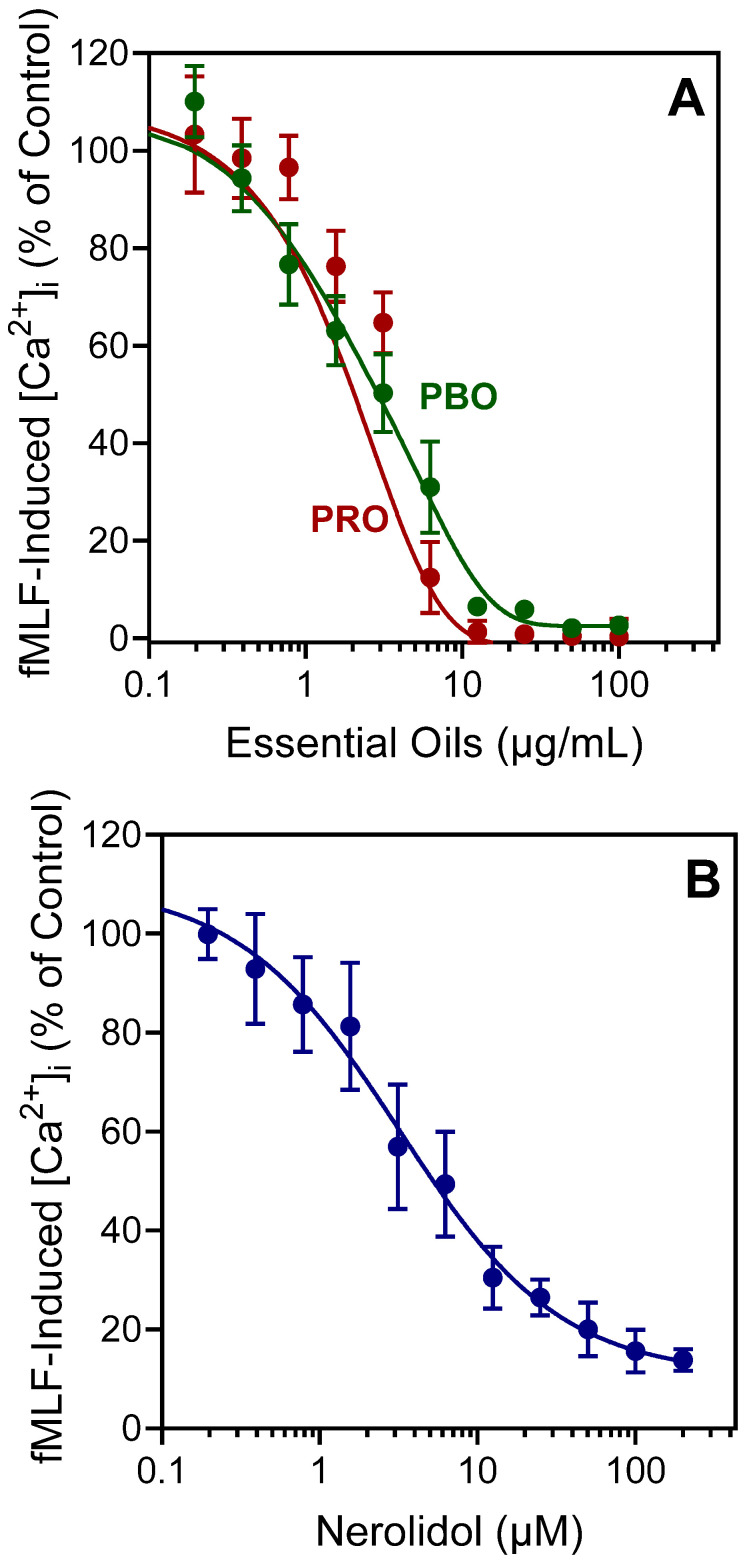
Effect of PBO, PRO, and nerolidol on *f*MLF-induced neutrophil [Ca^2+^]_i_. Human neutrophils were treated with the indicated concentrations of the essential oils (**A**), nerolidol (**B**), or 1% DMSO (negative control) for 10 min. The cells were then activated with 5 nM *f*MLF, and [Ca^2+^]_i_ was monitored as described. The data shown are presented as the mean ± SD from one experiment that is representative of three independent experiments with similar results.

It is well recognized that agonists can down-regulate neutrophil responses to subsequent treatment with heterologous or homologous agonists [79]. Thus, whether PBO, PRO, or nerolidol could alter agonist-induced [Ca^2+^]_i_ in human neutrophils stimulated with N-formyl chemotactic peptide was evaluated. As shown in Table 2 and Figure 2A, pretreatment with either PBO or PRO inhibited [Ca^2+^]_i_ in *f*MLF- and WKYMVM-stimulated neutrophils, with IC_50_ values in the micromolar range. Likewise, nerolidol pretreatment also potently inhibited *f*MLF- and WKYMVM-stimulated neutrophil [Ca^2+^]_i_ (Table 2). A representative, dose-dependent response for the inhibition of *f*MLF-induced neutrophil [Ca^2+^]_i_ by nerolidol is shown in Figure 2B.

Previously, several of the compounds that are also present in PBO and PRO (i.e., α-pinene, β-pinene, limonene, 1,8-cineole, myrcene, sabinene, and terpinolene; see Table 1) were shown to have no inhibitory effect on human neutrophil Ca^2+^ influx [34,35], whereas minor components farnesene (0.2–0.7% in PRO and PBO) and germacrene D (0.2% in PRO) were found previously to inhibit agonist-induced activation of human neutrophils [32,48] and, thus, could contribute to the inhibitory activity observed for PBO and PRO.

### 2.3. Effect of PBO, PRO, and Nerolidol on Neutrophil Chemotaxis

**Figure 3:**
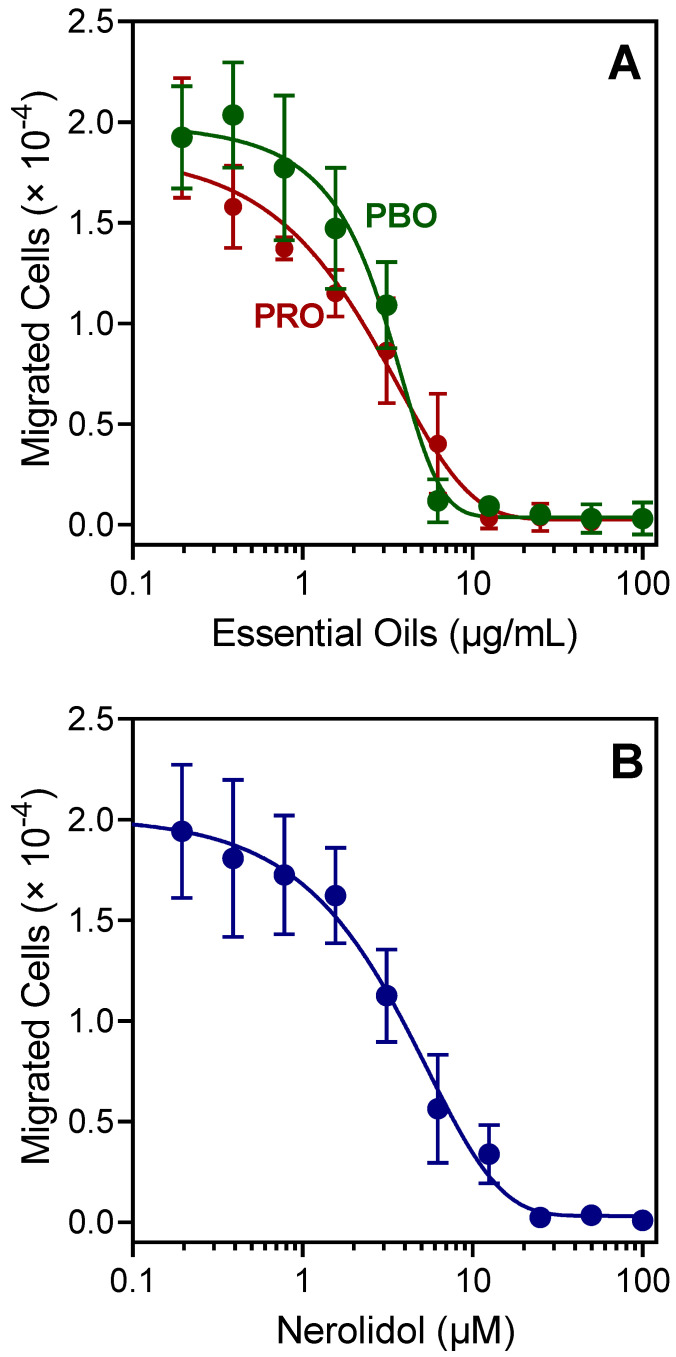
Effect of the PBO, PRO, and nerolidol on human neutrophil chemotaxis. Neutrophils were pretreated with the indicated concentrations of the essential oils (**A**) or nerolidol (**B**), and neutrophil migration toward 1 nM *f*MLF was measured, as described. The data are from one experiment that is representative of three independent experiments.

Various essential oils and their components were reported previously to inhibit neutrophil chemotaxis, including farnesene and germacrene D, which are minor components of PBO and PRO (Table 1). In the present study, the effects of PBO and PRO on human neutrophil chemotaxis were evaluated. Pretreatment with these essential oils dose-dependently inhibited *f*MLF-induced neutrophil chemotaxis (IC_50_ = 1.5 and 2.9 µg/mL, respectively) (Figure 3A). Likewise, pretreatment with nerolidol also inhibited *f*MLF-induced human neutrophil chemotaxis (IC_50_ = 3.9 µM) (Figure 3B).

**Figure 4:**
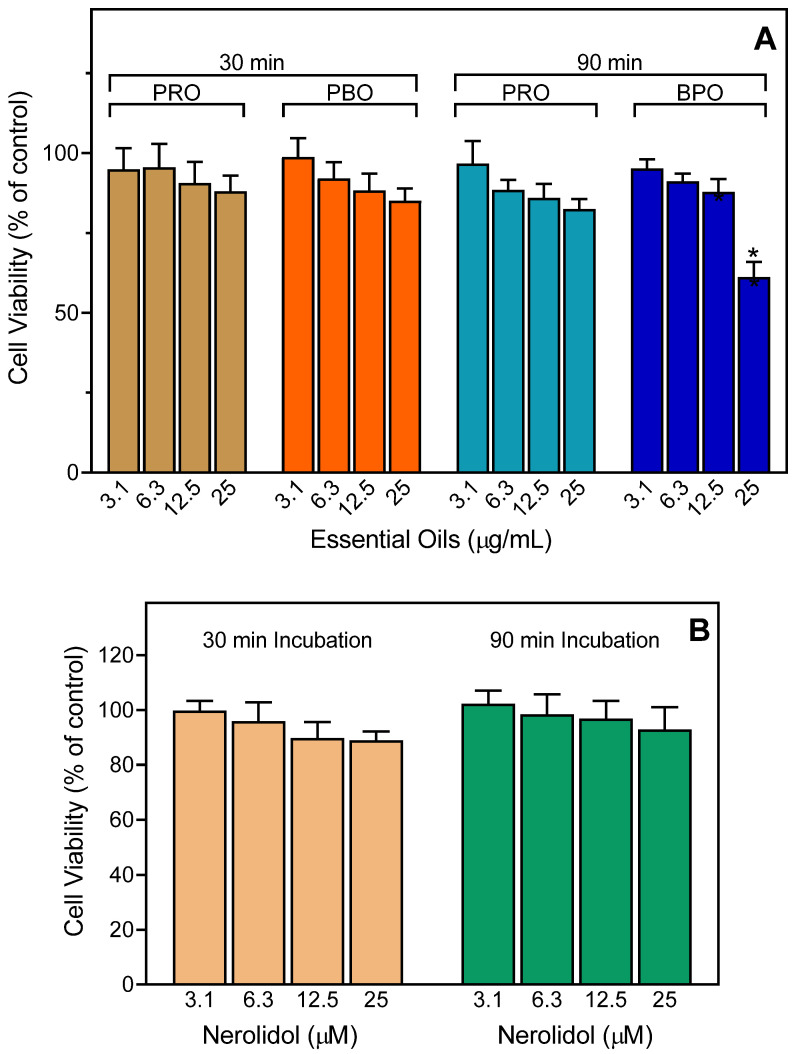
Evaluation of the cytotoxicity of PRO, PBO, and nerolidol. Human neutrophils were preincubated with the indicated concentrations of essential oil (**A**) or pure nerolidol (**B**) for 30 min or 90 min, and cell viability was analyzed, as described. Values are the mean ± SD of triplicate samples from one experiment that is representative of three independent experiments with similar results. * *p* < 0.05 compared to DMSO control.

To ensure that the effects of the essential oils and nerolidol were not influenced by possible toxicity, we evaluated the cytotoxicity of the PBO and PRO (up to 25 µg/mL) and pure nerolidol at various concentrations (up to 25 µM) in human neutrophils. We found that the PRO had little to no cytotoxicity at concentrations up to 25 µg/mL during 30- and 90-min incubation periods, which covers the times used to measure Ca^2+^ influx (up to 30 min) and cell migration (up to 90 min), although PBO had some cytotoxicity at the highest concentration (25 µg/mL) (Figure 4A). Note that the inhibitory effects of PBO on neutrophil functional activity were found at much lower concentrations (1–10 µg/mL) (see Figure 2 and Figure 3). Consistent with these results, nerolidol had no cytotoxicity for neutrophils at all concentrations and times tested (Figure 4B).

### 2.4. Identification of Potential Protein Targets for Nerolidol

The application of nerolidol is widespread across different industries, and it has been used in cosmetics and non-cosmetic products [80,81]. In fact, the U.S. Food and Drug Administration (FDA) has also permitted the use of nerolidol as a food flavoring agent [82]. The fact that nerolidol is a common ingredient in many products has attracted researchers to explore more medicinal properties of nerolidol that may exert beneficial effects on human health (reviewed in [80]).

In addition to the immunomodulatory activity reported here, nerolidol was reported to exhibit a number of biological activities. For example, nerolidol has demonstrated anticholinesterase, antioxidant, antinociceptive, antibacterial, anti-parasite, anti-inflammatory, and anxiolytic activities, suggesting it may be a promising phytochemical for the development of therapeutic drugs (reviewed in [63,80,83]). Likewise, nerolidol was effective against schistosomiasis [84] and babesiosis [85]. Nerolidol was also reported to have neuroprotective properties, presumably through its anti-inflammatory and antioxidant activities [86,87]. Likewise, nerolidol reduced adjuvant arthritis by down-regulating proinflammatory cytokines and up-regulating anti-inflammatory cytokines [88]. It also attenuated hypertension-induced hypertrophy in spontaneously hypertensive rats through modulation of insulin-like growth factor receptor II (IGF-IIR) signaling [89]. This acyclic sesquiterpene induced apoptosis via phosphatidylinositol-3-kinase (PI3K) and c-Jun N-terminal kinase (JNK) regulation through cell cycle arrest in MG-63 osteosarcoma cells [90]. Finally, changes in tissue myeloperoxidase concentrations, neutrophil and macrophage mRNA expression of monocyte chemoattractant protein-1 (MCP-1), and proinflammatory cytokine content (interleukin (IL)-1β, IL-6, and tumor necrosis factor (TNF)) protein and mRNA levels were significantly reduced by nerolidol [91].

The most important physicochemical parameters of nerolidol were calculated using SwissADME [92]. These parameters were identical for *E*/*Z* isomers, with the exception of a slight difference in iLogP values (Table 3). SwissADME also predicted that nerolidol can permeate the blood–brain barrier (BBB). In addition, nanotechnology using nerolidol-loaded nanospheres may also improve passage through the BBB [93].

**Figure 5:**
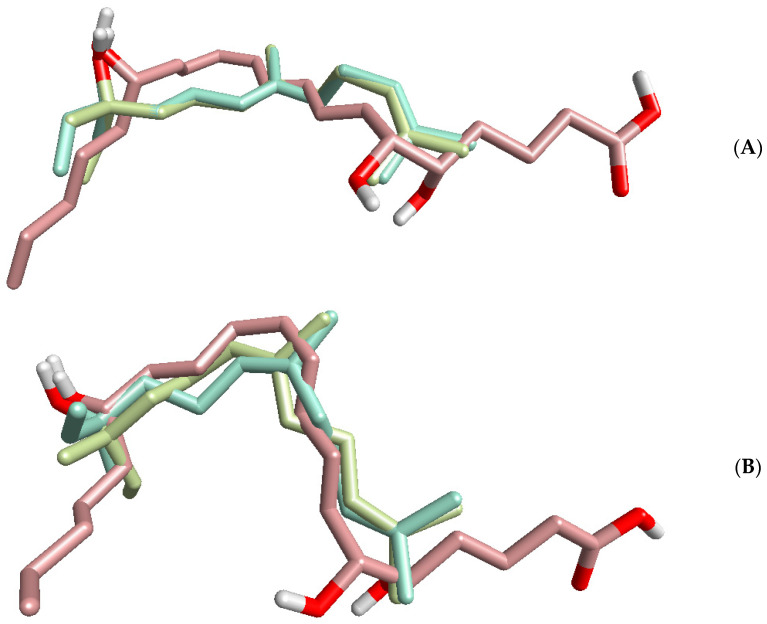
Alignments of lipoxin A_4_ with *E*-nerolidol (**A**) and *Z*-nerolidol (**B**). Alignments were performed using FieldTemplater software. Lipoxin A_4_ molecule is shown with a pink skeleton; the *R* and *S* enantiomers of nerolidol are shown with khaki and green skeletons, respectively.

Nerolidol is a lipophilic molecule (Table 3). Thus, the neutrophil signaling inhibitory mechanisms may be based on allosteric interactions of the nerolidol chain with the membrane portion of a receptor, and this issue mechanism is currently being investigated. Indeed, other lipophilic compounds, such as the bile acids deoxycholic acid and chenodeoxycholic acid, were reported to antagonize FPR1 at high concentrations (>100 µM) [94,95,96]. Moreover, lipoxin A_4_ was also reported as an allosteric modulator of the CB1 cannabinoid receptor and FPR2 [97,98]. To further investigate this issue, lipoxin A_4_ was aligned with the *E*- and *Z*-nerolidol enantiomers using FieldTemplater software (Figure 5). The results demonstrated that the alignments were governed mainly by the hydrophobic hydrocarbon skeletons of the compounds. The combined similarity measure of the superimpositions were relatively high (S = 0.690 and 0.678 for the or *Z* and *E* isomers, respectively; each of the superimpositions was obtained with two enantiomers of nerolidol aligned onto lipoxin A_4_), suggesting that nerolidol might mimic lipoxin A_4_ and maybe other related specialized pro-resolving mediators, including resolvins, maresins, and protectins [99]. Indeed, many of these molecules were demonstrated to act allosterically on a number of GPCRs (reviewed in [100]). Interestingly, we found previously that other compounds structurally similar to nerolidol (i.e., 6-methyl-3,5-heptadien-2-one (MHDO), geranylacetone, and farnesene) also inhibited agonist-induced neutrophil activity [34,35,48]. Although molecular targets for MHDO and farnesene were not identified, we showed that geranylacetone is a TRPV1 agonist [34].

Reverse-pharmacophore mapping [101] of the molecular structures of *E*/*Z*-nerolidol in the *R* and *S* enantiomer forms of each geometric isomer was performed to identify potential biological targets. PharmMapper compared a database of pharmacophore patterns with these compounds and generated target information, such as pharmacophoric characteristics and normalized fitness scores. Note, however, that PharmMapper depends on the availability of structures for pharmacophore mapping, and most GPCRs are not represented in the database.

The proper optical isomers of the compounds were submitted to the PharmMapper server, as mapping explicitly accounts for the three-dimensional structure of a molecule. The ten top-ranked potential targets found by PharmMapper are shown in Table 4, and this analysis indicated that kinases could be among the potential targets for nerolidol. Among the top ten ranked kinase targets for the nerolidol were extracellular signal-regulated kinase 2 (ERK2), mitogen-activated protein kinase (MAPK) activated protein kinase 2 (MAPKAPK2), proto-oncogene serine/threonine-protein kinase (Pim-1), c-Jun N-terminal kinase 3 (JNK3), and vascular endothelial growth factor receptor 2 (VEGFR2), a tyrosine kinase (Table 4).

MAPK signaling plays an important role in neutrophil signal transduction cascades [102], and studies have shown that members of the MAPK, JNK, and the ERK families of proteins are activated in response to neutrophil priming/activation (reviewed in [103]). It is also clear from previous studies that one or more of these MAPK pathways is induced by FPR [104,105,106]. Thus, nerolidol may be a general inhibitor of neutrophil activation through GPCRs, and further studies are in progress to evaluate this idea and identify the specific molecular targets.

## 3. Materials and Methods

### 3.1. Material

Plant material was collected from wild *P. balsamifera* trees in May 2022 along Bozeman Creek near Bozeman, MT, USA (45.582238° N, 111.022036° E). Botanical identification of the plant material was performed by botanist Robyn A. Klein from Montana State University (Bozeman). Propolis samples were collected during September 2021 from *Apis mellifera* hives located approximately 10 miles northwest of Bozeman (45.729971° N, 111.234961° E).

### 3.2. Materials

Dimethyl sulfoxide (DMSO), *N*-formyl-Met-Leu-Phe (*f*MLF), Trp-Lys-Tyr-Val-Met (WKYMVM), nerolidol (as mixture of *E*- and *Z*-isomers) and Histopaque 1077 were purchased from Sigma-Aldrich Chemical Co. (St. Louis, MO, USA). *n*-Hexane was purchased from Merck (Darmstadt, Germany). Fluo-4AM was purchased from Invitrogen (Carlsbad, CA, USA). Roswell Park Memorial Institute (RPMI) 1640 medium was purchased from HyClone Laboratories (Logan, UT, USA). Fetal calf serum and fetal bovine serum were purchased from ATCC (Manassas, VA, USA). Hanks’ balanced salt solution (HBSS; 0.137 M NaCl, 5.4 mM KCl, 0.25 mM Na_2_HPO_4_, 0.44 mM KH_2_PO_4_, 4.2 mM NaHCO_3_, 5.56 mM glucose, and 10 mM HEPES, pH 7.4) was purchased from Life Technologies (Grand Island, NY, USA). HBSS without Ca^2+^ and Mg^2+^ was designated as HBSS^–^; HBSS containing 1.3 mM CaCl_2_ and 1.0 mM MgSO_4_ was designated as HBSS^+^.

### 3.3. Essential Oil Extraction

Essential oil was obtained by hydrodistillation of *P. balsamifera* buds using a Clevenger-type apparatus, as previously described [35]. Crude propolis (100 g) was divided into small pieces and extracted by the same procedure. Conditions accepted by the European Pharmacopoeia (European Directorate for the Quality of Medicines, Council of Europe, Strasbourg, France, 2014) were used to avoid artifacts. Yields of the essential oils were calculated based on the amount of air-dried plant material used. Stock solutions of the essential oils were prepared in DMSO (10 mg/mL) for biological evaluation and in *n*-hexane (10% *w*/*v*) for gas chromatographic analysis.

### 3.4. Gas Chromatography–Flame Ionization Detector (GC-FID) and Gas Chromatography–Mass Spectrometry (GC-MS) Analysis

GC-MS analysis was performed with an Agilent 5975 GC-MSD system (Agilent Technologies, Santa Clara, CA, USA), as reported previously [107]. An Agilent Innowax FSC column (60 m × 0.25 mm, 0.25 μm film thickness) was used with He as the carrier gas (0.8 mL/min). The GC oven temperature was kept at 60 °C for 10 min, increased to 220 °C at a rate of 4 °C/min, kept constant at 220 °C for 10 min, and then increased to 240 °C at a rate of 1 °C/min. The split ratio was adjusted to 40:1, and the injector temperature was 250 °C. MS spectra were monitored at 70 eV with a mass range of 35 to 450 *m*/*z*. GC analysis was performed on an Agilent 6890N GC system. To obtain the same elution order as with GC-MS, the line was split for FID and MS detectors, and a single injection was performed using the same column and appropriate operational conditions. FID temperature was 300 °C. The essential oil components were identified by co-injection with standards (whenever possible), which were purchased commercially or isolated from natural sources. In addition, compound identities were confirmed by comparison of their mass spectra with those in the Wiley GC/MS Library (Wiley, NY, USA), MassFinder software 4.0 (Dr. Hochmuth Scientific Consulting, Hamburg, Germany), Adams Library, and NIST Library. Confirmation was also achieved using the in-house “Başer Library of Essential Oil Constituents” database obtained from chromatographic runs of pure compounds performed with the same equipment and conditions. A C_8_–C_40_ *n*-alkane standard solution (Fluka, Buchs, Switzerland) was used to spike the samples for the determination of relative retention indices (RRI). Relative percentage amounts of the separated compounds were calculated from the FID chromatograms.

### 3.5. Isolation of Human Neutrophils

For isolation of human neutrophils, blood was collected from healthy donors in accordance with a protocol approved by the Institutional Review Board at Montana State University (protocol #MQ041017). Neutrophils were purified from the blood using dextran sedimentation, followed by Histopaque 1077 gradient separation and hypotonic lysis of red blood cells, as described previously [108]. Isolated neutrophils were washed twice and resuspended in HBSS^–^. Neutrophil preparations were routinely >95% pure, as determined by light microscopy, and >98% viable, as determined by trypan blue exclusion. Neutrophils were obtained from multiple different donors; however, the cells from different donors were never pooled during experiments.

### 3.6. Ca^2+^ Mobilization Assay

Changes in intracellular Ca^2+^ concentrations ([Ca^2+^]_i_) were measured with a FlexStation 3 scanning fluorometer (Molecular Devices, Sunnyvale, CA, USA). Briefly, human neutrophils were suspended in HBSS^−^, loaded with Fluo-4AM at a final concentration of 1.25 μg/mL, and incubated for 30 min in the dark at 37 °C. After dye loading, the cells were washed with HBSS^−^, resuspended in HBSS^+^, separated into aliquots, and loaded into the wells of flat-bottom, half-area-well black microtiter plates (2 × 10^5^ cells/well). To assess the direct effects of test compounds or pure essential oils on Ca^2+^ influx, the compound/oil was added to the wells (final concentration of DMSO was 1%), and changes in fluorescence were monitored (λ_ex_ = 485 nm, λ_em_ = 538 nm) every 5 s for 240 s at room temperature after addition of the test compound. To evaluate the inhibitory effects of the compounds on *N*-formyl peptide receptor 1/2 (FPR1/FPR2) dependent Ca^2+^ influx, the compound or essential oil was added to the wells (final concentration of DMSO was 1%) with human neutrophils. The samples were preincubated for 10 min, followed by the addition of 5 nM *f*MLF or 5 nM WKYMVM. The maximum change in fluorescence, expressed in arbitrary units over baseline, was used to determine the agonist response. Responses were normalized to the response induced by 5 nM *f*MLF or 5 nM WKYMVM, which were assigned as 100%. Curve fitting (at least five or six points) and calculation of median effective concentration values (EC_50_ or IC_50_) were performed by nonlinear regression analysis of the dose–response curves generated using Prism 9 (GraphPad Software, Inc., San Diego, CA, USA).

### 3.7. Chemotaxis Assay

Human neutrophils were resuspended in HBSS^+^ containing 2% (*v*/*v*) heat-inactivated fetal bovine serum (2 × 10^6^ cells/mL), and chemotaxis was analyzed in 96-well ChemoTx chemotaxis chambers (Neuroprobe, Gaithersburg, MD, USA). In brief, neutrophils were preincubated with the indicated concentrations of the test sample (essential oil or pure compound) or DMSO (1% final concentration) for 30 min at room temperature and added to the upper wells of the ChemoTx chemotaxis chambers. The lower wells were loaded with 30 µL of HBSS^+^ containing 2% (*v*/*v*) fetal bovine serum and the indicated concentrations of test sample, DMSO (negative control), or 1 nM *f*MLF as a positive control. Neutrophils were added to the upper wells and allowed to migrate through the 5.0 µm pore polycarbonate membrane filter for 60 min at 37 °C and 5% CO_2_. The number of migrated cells was determined by measuring ATP in lysates of transmigrated cells using a luminescence-based assay (CellTiter-Glo; Promega, Madison, WI, USA), and chemiluminescence measurements were converted to absolute cell numbers by comparison of the values with standard curves obtained with known numbers of neutrophils. Curve fitting (at least eight to nine points) and calculation of effective concentration values (IC_50_) were performed by nonlinear regression analysis of the dose–response curves generated using GraphPad Prism 9 (San Diego, CA, USA).

### 3.8. Cytotoxicity Assay

Cytotoxicity of essential oils and pure nerolidol in human neutrophils was analyzed with a CellTiter-Glo Luminescent Cell Viability Assay Kit (Promega) according to the manufacturer’s protocol. Briefly, human neutrophils were cultured at a density of 10^4^ cells/well with different concentrations of essential oil or nerolidol (final concentration of DMSO was 1%) for 30 and 90 min at 37 °C and 5% CO_2_. Following treatment, substrate was added to the cells, and the samples were analyzed with a Fluoroscan Ascent FL microplate reader.

### 3.9. Molecular Modeling

PharmMapper [109] was used to identify potential protein targets for four possible geometric and optical isomers of nerolidol. PharmMapper recognizes potential targets based on “invert” pharmacophore mapping. The protein biotargets are represented by sets of pharmacophore points in reference databases incorporated in the software. PubChem (https://pubchem.ncbi.nlm.nih.gov) and ChemSpider (https://www.chemspider.com/) databases (accessed on 25 August 2022) were used as sources of initial 3D structures of our compounds: (3*R*,6*E*)-nerolidol (PubChem CID: 11241545), (3*S*,6*E*)-nerolidol (PubChem CID: 5281525), (3*S*,6*Z*)-nerolidol (ChemSpider ID: 21427544). The 3D structure of (3*R*,6*Z*)-nerolidol was obtained by a mirror reflection of the corresponding *S*-enantiomer using ChemOffice 2016 software. The 3D structures were saved in SDF format and uploaded into PharmMapper. The system automatically generated up to 300 conformers of each compound based on the software option. Pharmacophore mapping was performed using the “Human Protein Targets Only” database, which contained 2241 targets. The top 250 potential targets were retrieved for each compound evaluated, and the potential targets were sorted by normalized fit score.

The physicochemical properties of nerolidol were computed using SwissADME (http://www.swissadme.ch; accessed on 1 September 2022).

Alignments of lipoxin-A_4_ and enantiomers of *Z*- and *E*-nerolidol were performed using FieldTemplater software (Cresset Group, Cambridgeshire, UK).

### 3.10. Statistical Analysis

One-way analysis of variance (ANOVA) was performed on the data sets, followed by Tukey’s pair-wise comparisons. Pair-wise comparisons with differences at *p* < 0.05 were considered statistically significant.

## 4. Conclusions

Analysis of the composition of essential oils extracted from *P. balsamifera* buds and propolis collected in Montana showed that they were enriched with nerolidol and its derivatives. Further analysis of the immunomodulatory activity of these essential oils and nerolidol showed that they activated human neutrophils and were able to inhibit FPR1/FPR2 agonist-induced neutrophil activation and chemotaxis, which might contribute to the reported anti-inflammatory activity and other pharmacological properties of these extracts. The biological effects of PBO and PRO might be attributable primarily to nerolidol or to the synergistic effects of nerolidol with other active constituents. However, to verify the key targets responsible for the immunomodulatory effects of nerolidol, further experimental investigation is needed.

## Figures and Tables

**Table 2:** The effect of nerolidol and essential oils from propolis and *P. balsamifera* buds on human neutrophil [Ca^2+^]_i_ and chemotaxis.

Source of Essential Oil or Pure Compound	Activation of [Ca^2+^]_i_	Inhibition of [Ca^2+^]_i_		
		*f*MLF- Induced	WKYMVM- Induced	*f*MLF-Induced Chemotaxis
	EC_50_ (μg/mL)	IC_50_ (μg/mL)		
*P. balsamifera*	10.5 ± 1.1	1.8 ± 0.6	9.4 ± 1.9	1.5 ± 0.5
Propolis	18.3 ± 3.7	3.4 ± 0.1	0.9 ± 0.3	2.9 ± 1.3
	**EC_50_ (μM)**	**IC_50_ (μM)**		
Nerolidol	0.8 ± 0.1	4.0 ± 1.7	3.7 ± 0.4	3.9 ± 1.3

Legend: EC_50_ and IC_50_ values were determined by nonlinear regression analysis of the dose–response curves as described under Materials and Methods. The data are presented as the mean ± SD of three independent experiments.

**Table 3:** Chemical structures and physicochemical properties of nerolidol isomers according to SwissADME results.

Property	*E*-Nerolidol	*Z*-Nerolidol
	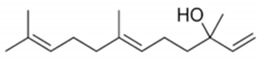	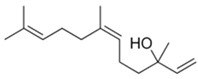
Formula	C_15_H_26_O	C_15_H_26_O
M.W.	222.37	222.37
Heavy atoms	16	16
Fraction Csp^3^	0.6	0.6
Rotatable bonds	7	7
H-bond acceptors	1	1
H-bond donors	1	1
MR	74.0	74.0
tPSA	20.23	20.23
iLogP	3.64	3.60
BBB permeation	Yes	Yes

Abbreviations: M.W., molecular weight (g/mol); MR, molar refractivity; tPSA, topological polar surface area (Å^2^); iLogP, lipophilicity; BBB, blood–brain barrier.

**Table 4:** Potential protein targets for nerolidol isomers identified by PharmMapper.

Rank	PDB ID	Target Name	Fit Score	Rank	PDB ID	Target Name	Fit Score
*R*-(*E*)-Nerolidol				*S*-(*E*)-Nerolidol			
1	1J96	AKR1C2	2.999	1	1P49	Steryl-sulfatase	2.987
2	1E7E	Serum albumin	2.997	2	3BMP	BMP2	2.983
3	1L6L	Apo A-II	2.991	3	2JBP	MAPKAPK2	2.981
4	1P49	Steryl-sulfatase	2.989	4	3DEJ	Caspase-3	2.979
5	3BMP	BMP2	2.982	5	2Q11	β-Secretase 1	2.977
6	2PIN	NR1A2	2.978	6	2PIN	NR1A2	2.977
7	3BGP	Pim-1	2.970	7	1BM6	Stromelysin-1	2.970
8	1PME	ERK2	2.966	8	1L6L	Apo A-II	2.969
9	1III	Transthyretin	2.962	9	1PME	ERK2	2.965
10	1TG6	CLPP	2.960	10	1QKU	Estrogen receptor	2.961
*R*-(*Z*)-Nerolidol				*S*-(*Z*)-Nerolidol			
1	1P49	Steryl-sulfatase	2.998	1	1P49	Steryl-sulfatase	3.000
2	2JBP	MAPKAPK2	2.991	2	3BGP	Pim-1	2.993
3	3CJF	VEGFR2	2.989	3	1F86	Transthyretin	2.992
4	3CGF	JNK3	2.989	4	1L6L	Apo A-II	2.991
5	3BMP	BMP2	2.983	5	2PG2	KIF11	2.987
6	2PIN	NR1A2	2.980	6	3BMP	BMP2	2.982
7	1SHJ	Caspase-7	2.970	7	1IF4	CA2	2.975
8	1YA8	LCE1	2.966	8	1J96	AKR1C2	2.974
9	1PME	ERK2	2.965	9	1OJ9	MAO-B	2.969
10	2O65	Pim-1	2.964	10	2JBP	MAPKAPK2	2.964

Abbreviations: AKR1C2, aldo-keto reductase family 1 member C2; Apo A-II, apolipoprotein A-II; BMP2, bone morphogenetic protein 2; CA2, carbonic anhydrase 2; CLPP, ATP-dependent Clp protease proteolytic subunit, mitochondrial; ERK2, extracellular signal-regulated kinase 2; JNK3, c-Jun N-terminal kinase 3; KIF11, kinesin family member 11; LCE1, liver carboxylesterase 1; MAO-B, monoamine oxidase B; MAPKAPK2, MAP kinase-activated protein kinase 2; NR1A2, thyroid hormone receptor β; Pim-1, proto-oncogene serine/threonine-protein kinase; VEGFR2, vascular endothelial growth factor receptor 2.

## Data Availability

Data are contained within the article.

## Sample Availability

Samples of essential oils are available from the authors.
